# Topical Spilanthol Inhibits MAPK Signaling and Ameliorates Allergic Inflammation in DNCB-Induced Atopic Dermatitis in Mice

**DOI:** 10.3390/ijms20102490

**Published:** 2019-05-20

**Authors:** Wen-Chung Huang, Chun-Hsun Huang, Sindy Hu, Hui-Ling Peng, Shu-Ju Wu

**Affiliations:** 1 Graduate Institute of Health Industry Technology, Research Center for Food and Cosmetic Safety, College of Human Ecology, Chang Gung University of Science and Technology, Taoyuan City 33303, Taiwan; wchuang@mail.cgust.edu.tw (W.-C.H.); hlpeng@mail.cgust.edu.tw (H.-L.P.); 2 Division of Allergy, Asthma, and Rheumatology, Department of Pediatrics, Chang Gung Memorial Hospital, Linkou, Taoyuan City 33303, Taiwan; 3 Department of Cosmetic Science, Research Center for Food and Cosmetic Safety, and Research Center for Chinese Herbal Medicine, College of Human Ecology, Chang Gung University of Science and Technology, Guishan Dist., Taoyuan City 33303, Taiwan; chuang@mail.cgust.edu.tw (C.-H.H.); sindyhu@hotmail.com (S.H.); 4 Department of Dermatology, Aesthetic Medical Center, Chang Gung Memorial Hospital, Taoyuan City 33303, Taiwan; 5 Department of Nutrition and Health Sciences, Research Center for Chinese Herbal Medicine, College of Human Ecology, Chang Gung University of Science and Technology, Taoyuan City 33303, Taiwan

**Keywords:** spilanthol, IgE, allergic inflammation, MAPK, atopic dermatitis

## Abstract

Atopic dermatitis (AD) is a recurrent allergic skin disease caused by genetic and environmental factors. Patients with AD may experience immune imbalance, increased levels of mast cells, immunoglobulin (Ig) E and pro-inflammatory factors (Cyclooxygenase, COX-2 and inducible NO synthase, iNOS). While spilanthol (SP) has anti-inflammatory and analgesic activities, its effect on AD remains to be explored. To develop a new means of SP, inflammation-related symptoms of AD were alleviated, and 2,4-dinitrochlorobenzene (DNCB) was used to induce AD-like skin lesions in BALB/c mice. Histopathological analysis was used to examine mast cells and eosinophils infiltration in AD-like skin lesions. The levels of IgE, IgG1 and IgG2a were measured by enzyme-linked immunosorbent assay (ELISA) kits. Western blot was used for analysis of the mitogen-activated protein kinase (MAPK) pathways and COX-2 and iNOS protein expression. Topical SP treatment reduced serum IgE and IgG2a levels and suppressed COX-2 and iNOS expression via blocked mitogen-activated protein kinase (MAPK) pathways in DNCB-induced AD-like lesions. Histopathological examination revealed that SP reduced epidermal thickness and collagen accumulation and inhibited mast cells and eosinophils infiltration into the AD-like lesions skin. These results indicate that SP may protect against AD skin lesions through inhibited MAPK signaling pathways and may diminish the infiltration of inflammatory cells to block allergic inflammation.

## 1. Introduction

Common symptoms of atopic dermatitis (also known as atopic eczema) include itching, redness, and cracking skin. Pathological characteristics include dry, fragile skin as a result of epidermal defense dysfunction. Due to abnormal immune function, a variety of allergens are able to penetrate the skin, making it more prone to allergic reaction or inflammation [1]. In addition, atopic dermatitis (AD) is the product of a series of complex interactions of innate and adaptive immune responses and IgE-mediated allergies to various exogenous antigens [2]. Serious inflammation is a hallmark of acute AD lesions, and chronic AD lesions are characterized by lichenified fibrosis and epidermal thickening [3].

Studies have found that allergic reactions activate T helper (Th) cells, and that an imbalance between Th1 and Th2 cells causes AD [4]. Activation of Th2 cells leads to an allergic response producing IgE and IgG1, which in turn strengthen the immune response [5,6]. IgE has a high affinity for the IgE receptor expressed on the surface of mast cells; if IgE adheres to the mast cells, they are called sensitized cells. Mast cells are Th2-activated regulatory cells that release a lot of inflammatory-related cytokines, which can cause inflammation and allergic reaction [7,8]. Th2-activated cells also enable the aggregation of eosinophils, causing localized severe inflammation. IgG2a production is dependent on Th1 cells, which can regulate the activity of Th2 cells. However, Th2 cells will inhibit the activity of Th1 cells, creating an imbalance in which Th2 cell activity is much higher than Th1 cell activity, which in turn can cause an allergic reaction. Th1 and Th2 immune response, AD, tend to Th2 and have allergic constitution [7,9,10]. Therefore, decreasing the activity of Th2 cells may improve skin symptoms of AD. In addition, MAPKs pathway, which include the extracellular signal-regulated kinase (ERK), c-jun N-terminal kinase (JNK), and p38 MAPK, have also been implicated in inflammatory signaling cascades. Phosphorylation of MAPKs causes the inflammatory mediators’ production and promotes an allergic inflammatory response. MAPKs are important pathways in the inhibition of allergic inflammation. Therefore, inactivation of MAPKs subsequently decreases the allergic inflammatory response [11,12,13].

*Spilanthes acmella* Murr. is used as traditional folk medicine to treat toothache in the East Asia area. It has demonstrated a variety of biological effects, including anesthesia, analgesia, diuretic, and antibacterial effects [14,15,16,17]. Interestingly, research has supported the use of *Spilanthes* plant extract as a nutritional supplement and sweetener [18]. Alkamides are the most abundant phytochemicals present in *S. acmella*. Spilanthol (SP): ((2E,6Z,8E)-N-isobutylamide-2,6,8- decatrienamide) is a high-value bioactive compound and belongs to alkamides from *S. acmella* [19]. In addition, SP is also found in genus *Spilanthes*, including *Acmella brachyglossa*, *Acmella ciliate*, etc. [20,21]. SP reportedly has antibacterial, analgesic, and anti-wrinkle properties [22,23]. Previous studies showed that extract of *A. olerecea* is used in treatment of skin diseases including scabies and psoriasis, and used in anti-age applications (antiwrinkle cream) [20]. In a previous study, we found that SP exerts its anti-inflammatory activity by suppressing intercellular adhesion molecule 1 (ICAM-1) and COX-2 expression, and blocking the phosphorylated JNK signaling pathway [24]. However, it is not yet known about SP used in treatment of AD.

Therefore, in this study we evaluated the effects of SP on AD and sought to understand the mechanisms through which SP regulates allergic inflammation. Our findings indicate that SP reduces Th2-mediated infiltration by mast cells and eosinophils and decreases ear and dorsal skin thickness, and SP also inhibits COX-2 and iNOS expression by blocking MAPK pathways in mice with DNCB-induced AD.

## 2. Results

### 2.1. Spilanthol Attenuates Ear Swelling in BALB/c Mice with DNCB-Induced AD

**Figure 1:**
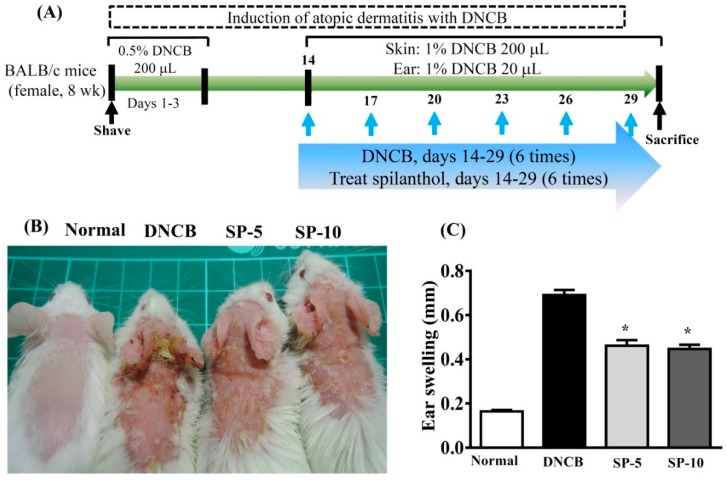
DNCB (2,4-dinitrochlorobenzene) induces atopic dermatitis (AD)-like lesions. (**A**) BALB/C mice were treated with 0.5% DNCB in acetone/olive oil (3:1) on days 1–3. Then, mice were challenged with 1% DNCB on days 14, 17, 20, 23, 26, and 29. AD-like lesions were treated with spilanthol (SP) (5 mg/kg or 10 mg/kg) or vehicle on days 14–27. Mice were sacrificed on day 30. (**B**) Clinical features of AD-like skin lesions treated topically with SP. (**C**) SP attenuates ear swelling by day 30 in DNCB-induced AD-like ear lesions. Data are presented as mean ± SEM (*n* = 8 mice/group). *p < 0.05, versus DNCB mice.

To investigate the effect of SP on AD, we used DNCB-induced ear and dorsal skin inflammation of BALB/c mice (Figure 1A). DNCB-induced AD-like symptoms included ear swelling, scarring, and excoriation of the skin and ear compared with normal mice (Figure 1B). We measured ear thickness on day 30 of DNCB-induced experimental model ear swelling. Topical administration of SP significantly reduced ear swelling compared with DNCB-sensitized mice (Figure 1C) (SP-5: 0.461 ± 0.25 mm, *p* < 0.05; SP-10: 0.44 ± 0.18 mm, *p* < 0.05, vs. the DNCB group: 0.69 ± 0.24 mm).

### 2.2. Spilanthol Attenuates Collagen Deposition and Reduces Epidermal and Dermal Thickness in BALB/c Mice with DNCB-Induced AD

**Figure 2:**
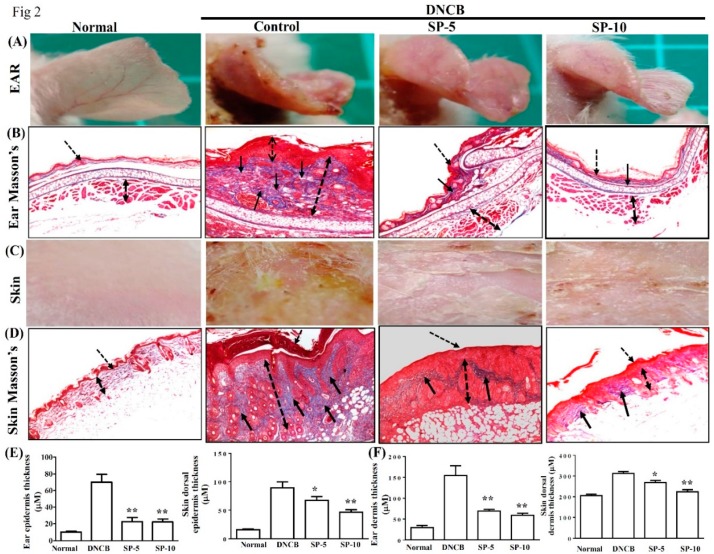
SP attenuated excessive dermal collagen and reduced epidermal thickness in DNCB-induced AD-like skin and ear lesions. (**A**) Ear lesions, (**B**) collagen deposition, (**C**) dorsal skin lesions, and (**D**) collagen deposition determined by Masson’s Trichrome staining, day 30. Black arrows: Collagen deposition; dashed arrows: Dermal hyperplasia. (**E**) Epidermal and (**F**) dermal thickness of ear and skin. Data presented as mean ± SEM (*n* = 8 mice/group). *p < 0.05, **p < 0.01 versus DNCB mice.

Masson’s Trichrome staining was used to evaluate collagen deposition and tissue fibrosis in DNCB-induced AD-like lesions. The main object of Masson’s Trichrome staining is collagen; collagen fibers were stained blue and the background was stained red. Hence, we used this staining to evaluate the improvement of collagen deposition after SP administration. Treatment with SP-5 or SP-10 significantly reduced ear thickness and hardening of the dorsal skin surface caused by inflammation; the remodeling on day 30 compared with the DNCB-sensitized group is shown in an image map (Figure 2A,C). Masson’s Trichrome stain revealed that at day 30, collagen deposition in AD-like skin lesions was significantly lower in the ear (Figure 2B) and dorsal skin (Figure 2D) of the SP-5 and SP-10 groups than in the DNCB-sensitized group. Thicknesses of both the epidermis and dermis were also significantly reduced in the ear (Figure 2E) and dorsal skin (Figure 2F) of SP-treated groups compared with the DNCB-sensitized group at day 30. Collectively, these results support that SP administration modulates the recovery of AD-like lesions by reducing epidermal and dermal hyperplasia, down-regulating collagen over-build.

### 2.3. Spilanthol Inhibits Mast Cell Infiltration and Affects Serum Cytokines in BALB/c Mice with DNCB-Induced AD

**Figure 3:**
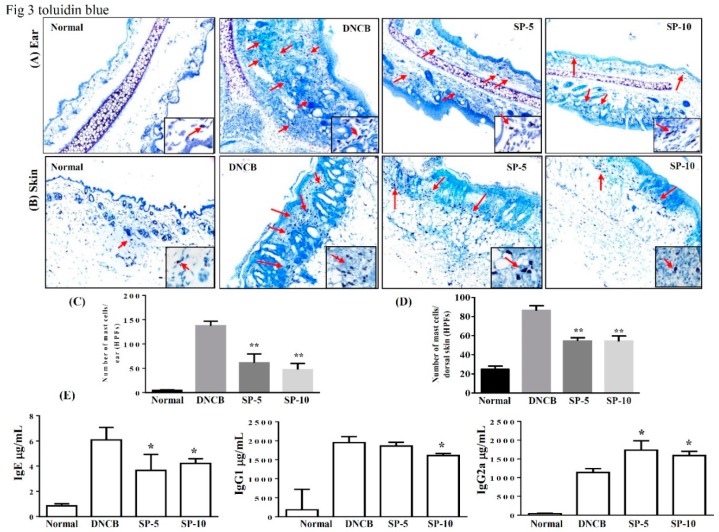
SP inhibits mast cell infiltration and modulates cytokine levels in DNCB-induced AD-like skin and ear lesions. Mast cell infiltration (red arrows) stained with toluidine blue in (**A**) ear and (**B**) dorsal skin sections. (**C**) Mast cells measured under 10–15 high-power fields (HPFs) in ear and (**D**) dorsal skin. (**E**) Serum levels of IgE, IgG1, and IgG2 measured using ELISA. Data presented as mean ± SEM (*n* = 8 mice/group). * *p* < 0.05, ** *p* < 0.01 versus DNCB mice.

Exposure to allergens stimulates IgE production in tissue and activates mast cells, then IgE and mast cells can induce complex immune responses and allergic symptoms [25]. Activated mast cells release inflammatory mediators, causing allergic inflammation in AD. To control activated mast cell release, inflammatory mediators can reduce allergic inflammation in AD [26,27]. Therefore, we focused on determining local infiltration by mast cells and assessing the inhibitory effect of SP on mast cell infiltration in mice with DNCB-induced AD. Toluidine blue staining of the ear and dorsal skin of DNCB-treated mice was performed to observe mast cell features. Topical administration of SP suppressed mast cell infiltration in the ears and dorsal skin compared with the DNCB-sensitized group (Figure 3A,B). The number of mast cells significantly decreased after SP administration compared with the DNCB-sensitized group (ear: SP-5: 61.5 ± 8.7, *p* < 0.01, SP-10: 47.2 ± 7.2, *p* < 0.01, vs. the DNCB-sensitized group: 137.4 ± 5.5; skin: SP-5: 54.6 ± 2.1, *p* < 0.01, SP-10: 53.2 ± 2.4, *p* < 0.01, vs. the DNCB-sensitized group: 87.8.0 ± 3.1) (Figure 3B,D). We also measured antibody levels to determine whether SP was able to modulate the allergic response in serum. Increasing serum IgE level is a major characteristic of AD, and we found that topical administration of SP significantly suppressed serum IgE and IgG1 levels in SP-10 mice, whereas serum IgG2a levels were significantly increased in SP-treated mice compared with the DNCB-sensitized group (Figure 3E). This suggests that SP can suppress the infiltration of mast cells and modulate the immune response.

### 2.4. Spilanthol Suppresses Eosinophil Infiltration and Inhibits Protein Expression of MAPK Signaling Pathways in BALB/c Mice with DNCB-Induced AD

**Figure 4:**
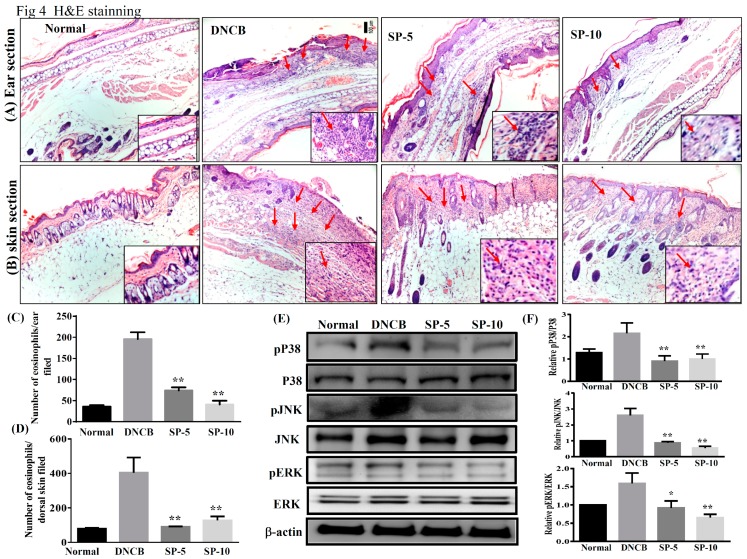
SP suppressed eosinophil infiltration and blocked mitogen-activated protein kinase (MAPK) signaling in DNCB-induced AD-like skin and ear lesions. Eosinophil infiltration (red arrows) in (**A**) ear and (**B**) dorsal skin lesions, determined by hematoxylin and eosin (H&E) stain. The number of eosinophils infiltrating the (**C**) ear and (**D**) dorsal skin measured under 10–15 high-power fields (HPFs). (**E**) Western blotting assays of p-extracellular signal-regulated kinase (p-ERK), p-p38, and p-c-jun N-terminal kinase (p-JNK) (*n* = 6/group), and (**F**) expression of p-ERK, p-p38, and p-JNK relative to ERK, p38, and JNK. The proteins were normalized to total JNK, ERK, and p38 protein levels, the total MAPK levels were used as internal controls. The relative intensity was calculated as the ratio of the intensities of the pP38, p-JNK, and p-ERK bands to the intensity of the total P38, JNK, and ERK bands, respectively. Data presented as mean ± SEM; *p < 0.05, **p < 0.01 versus DNCB mice. 100x magnification; amplified graph is 200x; *n* = 8 mice/group. Data presented as mean ± SEM. *p < 0.05, **p < 0.01 versus sensitized control mice.

To investigate the effect of SP on ear and dorsal skin, sections were stained with hematoxylin and eosin (H&E) to examine eosinophil infiltration in AD-like skin lesions. DNCB-sensitized mice exhibited more eosinophil infiltration than non-sensitized control mice. Increasing the permeability of blood vessels allows eosinophils to infiltrate into tissue. Topical administration of SP significantly decreased eosinophil infiltration compared with DNCB-sensitized mice (Figure 4A,B). The number of eosinophils decreased significantly after administration of SP compared with DNCB-sensitized mice (ear: SP-5: 74.5 ± 8.1, *p* < 0.01, SP-10: 40.7 ± 2.1, *p* < 0.01, vs. the DNCB group: 196.8.0 ± 4.8; dorsal skin: SP-5: 90.8 ± 4.3, *p* < 0.01, SP-10: 118.1 ± 2.6, *p* < 0.01, vs. the DNCB group: 403.5 ± 8.3) (Figure 4C,D).

The production of inflammation mediators by activated MAPK signaling pathways is also related to allergic inflammation. Therefore, we also investigated the effect of SP on the expression of ERK1/2, p38, and JNK proteins (Figure 4E). Results showed that levels of phosphorylated MAPK proteins (p-p38, p-JNK, and p-ERK) were increased significantly more in DNCB mice than in non-sensitized control mice, and topical administration of SP significantly decreased phosphorylation of ERK1/2, p38, and JNK in SP-treated mice compared with DNCB-sensitized mice (Figure 4F). These results indicated that SP suppressed allergic inflammation by blocking MAPK signaling pathways.

### 2.5. Spilanthol Inhibits the Expression of Pro-Inflammatory Factors COX-2 and iNOS in BALB/c Mice with DNCB-Induced AD

**Figure 5:**
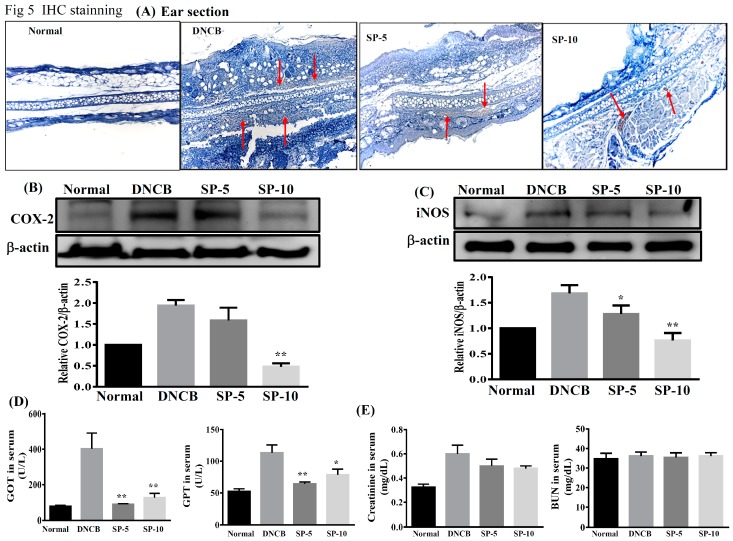
Effects of SP on cyclooxygenase-2 (COX-2) and inducible NO synthase (iNOS) in DNCB-induced AD-like skin lesions. (**A**) Immunohistochemical staining of COX-2 in ear (red arrows). (**B**) COX-2 level assayed by Western blot, and COX-2 protein expression relative to β-actin. (**C**) iNOS level assayed by Western blot, and iNOS protein expression relative to β-actin. Quantification of β-actin, iNOS, and COX-2 expression. β-actin expression was used as an internal control, the relative intensity was calculated as the ratio of the intensities of the COX-2 and iNOS bands to the intensity of the β-actin. Effects of spilanthol on (**D**) serum GOT and GPT, and (**E**) serum creatinine and BUN. Serum was centrifuged, collected, and evaluated by ELISA. Data presented as mean ± SEM; *p < 0.05, **p < 0.01 versus DNCB mice. 100x magnification; amplified graph is 200x; *n* = 8 mice/group. Data presented as mean ± SEM. * *p* < 0.05, ** *p* < 0.01 versus sensitized control mice.

Studies have indicated that increases in pro-inflammatory factors COX-2 and iNOS are observed in patients with AD [28]. Next, we investigated the effect of SP on DNCB-induced COX-2 and iNOS expression in AD-like mice. Immunohistochemistry showed that expression of COX-2 was lower in paraffin sections of ear biopsies (Figure 5A). Immunoblot analysis revealed that the levels of COX-2 and iNOS were significantly lower in SP-treated groups than in DNCB-sensitized mice (Figure 5B,C). Immunoblot analysis of COX-2 expression was consistent with the results of the immunohistochemical analysis. These results demonstrate that SP can reduce the inflammatory response by down-regulating the expression of inflammatory mediators COX-2 and iNOS in AD-like mice. We also evaluated the effects of SP on liver and kidney toxicity, as demonstrated by the drastic elevation of serum glutamate-oxaloacetic transaminase (GOT), glutamate-pyruvate transaminase (GPT), creatinine, and blood urea nitrogen (BUN). We found that serum GOT and GPT in the four groups of mice were within the normal range, although SP significantly decreased serum GOT and GPT levels in SP-treated mice compared with DNCB-sensitized mice (Figure 5D). In addition, serum creatinine and BUN levels were statistically similar among all experimental groups (Figure 5E). In brief, SP does not injure the liver or kidneys of AD-like mice.

## 3. Discussion

In this study, we investigated the anti-AD activity of SP in BALB/c mice with DNCB-induced AD. DNCB is an allergenic chemical commonly used to induce AD in animal models [27,29]. Environmental or allergic AD is known as extrinsic type AD, and genetic or non-allergic AD is known as intrinsic type AD [30]. Extrinsic or environmental factors induce severe AD through stimulation, triggering IgE-mediated forms of skin inflammation and allergic reaction [31]. AD is one of the most common chronic inflammatory skin diseases, and is characterized by erythema, dry skin, pruritus, and abnormal immune responses [27]. Repeated triggering of the allergic-inflammatory response leads to remodeling and hardening of the skin surface, leading the epidermis to thicken and break, resulting in infiltration of eosinophils and mast cells in AD skin lesions [32]. We found that topical treatment with SP ameliorated DNCB-induced AD-like skin lesions and improved skin lesion severity, ear swelling, and epidermal thickness in DNCB-treated BALB/c mice (Figure 1 and Figure 2). Moreover, SP reduced DNCB-induced collagen hyperplasia in AD-like skin lesions, as determined by Masson’s stain (Figure 2B,D). These results suggest that SP may be useful for the treatment of AD by reducing hyperkeratosis and fibrotic remodeling of the skin.

In the pathogenesis of AD, epidermal barrier function is impaired and the infiltration of environmental allergens into the skin increases, which in turn causes the allergic reactions and inflammation that are major characteristics of IgE-mediated hypersensitivity reactions [33]. Studies have shown that excessive IgE levels are closely related to imbalances of Th1 and Th2 cells in AD patients [30]. Th2-related cytokines, including of IL-4, are stimulators of IgE synthesis; excessive levels of IgE will activate mast cells and IL-5 induces eosinophil differentiation and infiltration into AD skin lesions, causing allergic inflammation. Excessive secretion of cytokines by activated Th2 cells will contribute to AD symptoms; therefore, down-regulation of Th2 cytokines may decrease production of IgE and improve AD [27,34]. Studies have indicated that increased secretion of Th1-related cytokines and decreased Th2-related cytokine levels may prevent excessive IgE production [35]. Reportedly, the IgG1 immune complex is responsible for class-switching to Th2 cytokines, and IgG2a is oriented toward Th1 cytokine immune deviation [36]. In addition, adjusting the balance of Th1/Th2 cytokines to inhibit mast cell activation, then decreasing the levels of IgG1 and increasing IgG2a has a significant anti-allergic inflammatory effect [37]. We found that topical treatment with SP reduced IgE levels and regulated IgG1 and IgG2a levels in DNCB-treated BALB/c mice (Figure 3E). In addition, SP also improved the infiltration of mast cells and eosinophils into AD skin lesions (Figure 3A,B and Figure 4A,B). These findings suggest that SP has a significant anti-allergic effect in AD. The SP may suppress eosinophil infiltration and down-regulate IgE expression to reduce mast cell infiltration into AD skin lesions.

Skin epidermal barrier dysfunction causing hardening and fragility of the skin surface is one of the main causes of AD, and inflammation can be modulated to reduce skin barrier function, thus aggravating lesions [29,30]. In addition, studies have indicated that constituents of MAPK pathways, including ERK, JNK, and p38, are involved in the pathogenesis of AD; in other words, AD is a chronic allergic inflammatory skin disease [38]. MAPK pathway activation promotes a number of inflammatory mediators, including COX-2 and iNOS [39]. COX-2 catalyzes arachidonic acid into prostaglandin, the levels of prostaglandin and COX-2 activity are related to promote inflammatory pain [40]. iNOS is produced by cytokines in inflammatory cells, which generate the free radical NO from L-arginine. iNOS and NO are related to cellular oxidative stress and the host cellular immune response [41] Several reports have shown that SP exhibits anti-inflammatory efficacy in vitro and in vivo [18,20,21,22]. However, it has been unknown whether SP exhibits anti-inflammatory activity in AD skin lesions. DNCB is a potential allergen that can induce skin sensitivity and inflammation [42]. Therefore, we identified SP as a possible suppressor of MAPK signal pathways triggered by DNCB in mice with DNCB-induced AD. In this work, we found that the anti-allergic effects of SP seem to occur through the blocked phosphorylation of ERK1/2, JNK, and p38 MAPK signaling pathways, and suppress COX-2 and iNOS expression in DNCB-induced AD skin lesions (Figure 4 and Figure 5). Based on these results, we suggest that SP may be useful as a treatment for allergic inflammation in AD.

**Figure 6:**
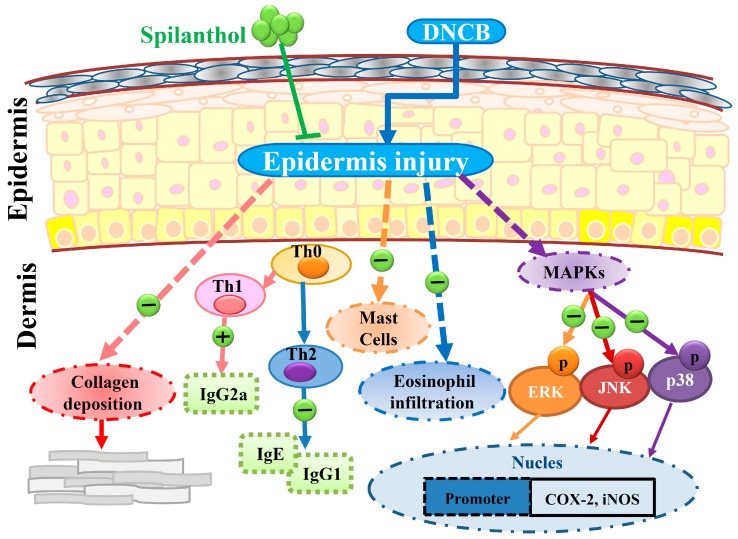
Topical spilanthol improves mast cell infiltration, modulates Th1/Th2 cytokine levels, and inhibits MAPK signaling, ameliorating allergic inflammation in DNCB-induced atopic.

We found that SP not only inhibited the levels of IgE and IgG2a, but also increased IgG1 level. SP may therefore be associated with mast cell-related allergic effects. Furthermore, SP significantly decreased eosinophil infiltration and reduced the expression of pro-inflammatory factors COX-2 and iNOS by suppressing MAPK pathways. SP may be associated with the improvement of eosinophil-related allergic inflammation in AD-like skin lesions. In addition, we found evidence that SP decreased collagen over-deposition in the dermis and reduced epidermal thickness in AD-like lesions. Collectively, we propose a model to explain the anti-allergic effects of SP in AD-like mice (Figure 6).

In summary, we present the first study demonstrating that SP has anti-AD potential. Results of this study demonstrate that SP can improve AD symptoms. SP-regulated Th1/Th2 balance, inhibited mast cell hyperplasia, and suppressed MAPK pathways ameliorated DNCB-induced AD-like skin inflammation in mice.

## 4. Materials and Methods

### 4.1. Animals

Eight-week-old female BALB/c mice were purchased from the National Laboratory Animal Center (Taiwan) and housed at the Animal Center of Chang Gung University in an air-conditioned room at a consistent temperature (23 ± 2 °C) and 55 ± 15% humidity, with a 12 h light–dark cycle. All procedures involving animals were approved in accordance with the guidelines and regulations of the Laboratory Animal Care Committee of Chang Gung University of Science and Technology and Chang Gung University (IACUC approval number: 2015-020; 29 December 2015).

### 4.2. DNCB Induction of AD-Like Skin Lesions and Spilanthol Treatment

Spilanthol was purchased from ChromaDex, Irvine, CA, USA. Mice were randomly divided into four groups (*n* = 8 per group): A mock-sensitized control group were sensitized and challenged with normal saline; a sensitized control group were treated with DNCB in a 3:1 ratio of acetone:olive oil; an SP-5 group were challenged with DNCB and treated topically with SP 5 g/kg; and an SP-10 group were challenged with DNCB and treated topically with SP 10 g/kg. The dorsal skin was shaved, and then sensitized using DNCB (Sigma-Aldrich, St. Louis, MO, USA) as described in a previous study [25]. To sensitize the skin, 200 µL 0.5% DNCB in acetone:olive oil (3:1) was applied to the shaved area on experimental days 1–3. Next, for the challenge process, 100 µL of 1% DNCB was applied to each ear and the dorsal skin on experimental days 14, 17, 20, 23, 26, and 29. All SP treatments were applied to the ears and backs of the mice daily on experimental days 14 to 29. The experimental design is described in Figure 1A.

### 4.3. Measurement of Ear and Epidermal Thickness

Images were captured weekly with a digital camera (Coolpix, Nikon Inc., Tokyo, Japan) to record clinical symptoms on the ear and dorsal skin. Ear thickness was measured using a dial gauge (Olympus, Tokyo, Japan) on day 30. Ear and skin epidermal thickness were measured using Masson’s stain and the aid of a microscope with Image-Pro Plus software (version 6.0 for Windows).

### 4.4. Histopathological Analysis

The ear and dorsal skin of each mouse were obtained on day 30 and fixed in 10% formalin. These tissues were cut into 6-µm thick sections and stained with Masson’s, hematoxylin and eosin (H&E), toluidine blue, and immunohistochemical stain, as previously described [25,39]. The sections were under 10–15 high-power fields (HPFs) using a light microscope at 100–200× magnification to measure mast cells and eosinophils. Mast cells and eosinophils were stained with toluidine blue and H&E, respectively. Collagen accumulation was assessed by Masson’s stain and using COX-2 antibody; immunohistochemical stain was used to observe positive COX-2 staining. Then, sections were examined using light microscopy to observe histological changes in all stained sections.

### 4.5. Measurement of Serum IgE and Cytokines

Blood samples were collected from BALB/c mice on day 30 and centrifuged at 3000 g for 10 min at 4 °C to obtain serum. The levels of serum IgE, IgG1, and IgG2a were measured using enzyme-linked immunosorbent assay (ELISA) kits (BD Biosciences, San Diego, CA, USA) according to the manufacturer’s instructions. Optical density was measured using a microplate reader (Multiskan FC, Thermo, Waltham, MA, USA). In addition, liver function indices (GPT and GOT) and kidney function indices (BUN and creatinine) were analyzed enzymatically using commercially available assay kits (Wako Pure Chemical, Osaka, Japan).

### 4.6. Western Blot Analysis

Dorsal skin samples were harvested on day 30 and stored at −80 °C. To investigate protein expression, protein lysates were prepared using protein lysis buffer (Sigma, St. Louis, MO, USA). The protein amounts were quantitated using the BCA protein assay kit (Pierce). Then, equal amounts of protein were separated on 10% SDS-PAGE gels and electrotransferred to polyvinylidene fluoride membranes (PVDF; Millipore, Billerica, MA, United States). The PVDF membranes were probed with primary antibodies raised against COX-2 and iNOS (Santa Cruz, CA, USA); ERK1/2, p38, JNK, phospho-ERK 1/2, phospho-p38, and phospho-JNK (Millipore); and β-actin (Sigma) overnight at 4 °C. Next, membranes were washed 3 times in Tris-buffered saline with Tween 20 (TBST) buffer (150 mM NaCl, 10 mM Tris-HCl pH 8.0, 0.1% Tween 20), then incubated in secondary antibodies at room temperature for 1 h, followed by incubation with HRP-conjugated secondary antibodies at room temperature for 1 h. Finally, the membranes were washed using TBST and incubated with Luminol/Enhancer Solution (Millipore). Protein bands were quantitated using the BioSpectrum 600 system (UVP, Upland, CA, United States).

### 4.7. Statistical Analysis

Data are reported as the mean ± standard error of the mean (SEM). All statistical significance was assessed using one-way analysis of variance (ANOVA) and Tukey’s test. Differences were considered statistically significant at *p* < 0.05.

## Abbreviations

AD	Atopic dermatitis
SP	Spilanthol
IgE	Immunoglobulin (Ig) E
IgG2a	Immunoglobulin (Ig) G2a
IgG1	Immunoglobulin (Ig) G1
COX-2	Cyclooxygenase-2
iNOS	Inducible NO synthase
DNCB	2,4-dinitrochlorobenzene
MAPK	Mitogen-activated protein kinase
ERK	Extracellular signal-regulated kinase
JNK	c-jun N-terminal kinase
